# TaqI polymorphism of the VDR gene: aspects related to the clinical behavior of COVID-19 in Cuban patients

**DOI:** 10.1186/s43042-021-00206-4

**Published:** 2021-11-30

**Authors:** Estela Morales Peralta, Yaíma Zúñiga Rosales, Teresa Collazo Mesa, Elvia Nelmi Santos González, Yadira Hernández Pérez, María de los Ángeles González Torres, Hilda Roblejo Balbuena, Beatriz Marcheco Teruel

**Affiliations:** 1 Facultad de Ciencias Médicas 10 de Octubre, Universidad de Ciencias Médicas de La Habana, 10 de Octubre CP 10500, Havana, Cuba; 2 National Center of Medical Genetics of Cuba, Havana, Cuba

**Keywords:** COVID-19, Genetic polymorphism, Cuba, Alleles, Vitamin D, Receptor

## Abstract

**Purpose:**

To determine the relationship between the genotypes of the TaqI polymorphism of *VDR* gene and the clinical forms of COVID-19 in Cuban patients.

**Methods:**

TaqI polymorphism was determined by the PCR in 104 Cuban patients, who suffered different clinical forms of COVID-19.

**Results:**

There was a greater possibility of presenting symptomatic forms [OR = 2.081, 95% CI: 0.243–17.842], even severe [OR = 1.200, 95% CI: 0.217–6.638], related to the tt genotype.

**Conclusion:**

There are signs of association between the risk of developing COVID-19 and the genotypes of the TaqI polymorphism of the *VDR* gene in the studied Cuban patients.

Dear Editor,

## Background

The recently described disease COVID-19, caused by the SARS-CoV-2 coronavirus infection, has a variable clinical behavior that ranges from asymptomatic to severe forms. The threat of developing severe clinical forms is related to risk factors such as: advanced age and the presence of comorbidities; [1] although unfavorable evolutions have been described in young and healthy people [2].

Gene polymorphisms are variations in DNA sequence that frequently appear in the population; they are often interpreted as benign or of unknown significance. [3] They have been associated with vulnerability, or resistance, to certain infectious agents [4].

Vitamin D acts on innate and adaptive immunity. It is linked to inflammation and the immune response and it presents an immunomodulatory, anti-inflammatory, antifibrotic and antioxidant effects. A concentration decrease has been observed associated with the risk of contracting viral infections of the respiratory tract and developing acute lung injuries [4, 5]; therefore, it is suggested that its deficiency may constitute a risk for developing a severe form of COVID-19 [6, 7].

The SARS-CoV-2 virus binds to receptors of the ACE2 in the airways of infected patients to enter host cells [8]. Vitamin D is involved in the expression of ACE2 in lung tissue [9].

There is evidence that polymorphisms in the gene that codes for the synthesis of Vitamin D receptor (VDR) are related to D hypovitaminosis [10]. VDR is a member of the family of nuclear receptors and the gene that encodes it is located at the 12q13.11 locus [11]. Codon 352 of its exon nine is polymorphic and it can exist as ATC or ATT. The resulting alleles are designated, according to the absence or presence of the restriction site of the TaqI enzyme, as T (wild-type) or t (mutated) [12]. This polymorphism has been described as a factor related to resistance to infectious diseases [13]; therefore, it also could possibly be related to COVID-19.

Knowing the human genetic variants linked to the predisposition to the development of COVID-19, and even its most severe clinical forms, will allow the design of prevention strategies that can contribute to generating a clinical guideline to treat this disease, based on advances in genomic medicine.

The purpose of this research was to determine the relationship between the genotypes of the TaqI polymorphism of *VDR* gene and the clinical forms of COVID-19 in Cuban patients.

## Methods

A sample of 104 patients was randomly taken from older than one-year-old Cuban citizens, without comorbidities related to TaqI polymorphism of the *VDR* gene (infectious diseases, cancer, leukemia, etc.) [13, 14], and with positive PCR (polymerase chain reaction) for SARS-CoV-2; that since June 11, 2020 were epidemiologically discharged. Their age was registered and taking into account the clinical manifestations during the disease, they were classified as: asymptomatic (cases in which infection progressed without symptoms or clinical signs), mildly and moderately symptomatic (clinical signs of infection and/or referred symptoms were detected without clinical complications), and severe symptomatic (requiring intensive clinical care, due to severe complications).

DNA was obtained from 4 mL of peripheral blood, collected in Vacutainer with EDTA (K3E 7.2 mg). The QIAsymphony DNA kits were used, according to the manufacturers' instructions [15]. The genotype of the TaqI polymorphism of the vitamin D receptor gene was determined by the PCR method designed by Taylor and his collaborators [14].

The data obtained were processed with the statistical program SPSS (Statistical Package for Social Sciences), version 22.0 to determine the risk of developing clinical forms of COVID-19. According to the genotype of the polymorphism studied, the odds ratio or OR was calculated with a 95% confidence interval.

This research was approved by the Research Ethics Committee of the National Center of Medical Genetics of Cuba. The principles for medical research on human beings included in the Declaration of Helsinki, as part of which the participants signed an informed consent, were followed [16].

## Results and discussion

Researches, like the one presented, have made it possible to identify polymorphisms related to predisposition, not only to present infectious diseases but also to develop forms with greater clinical severity [17]. This has proven to be very useful, especially for diseases like COVID-19, that have no specific treatment yet, and they are valuable to outline preventive measures.

According to clinical severity, the median age [with interquartile range] was as follows: in asymptomatic patients: 40.43 [12–68]; in mildly and moderately symptomatic ones: 43.80 [4–96] and in severe symptomatic: 62.04 [23–90].

In the most severe forms, the average age was higher, as has been described, possibly related to immune-senescence and the increased probability of presenting comorbidities [18].

**Fig. 1:**
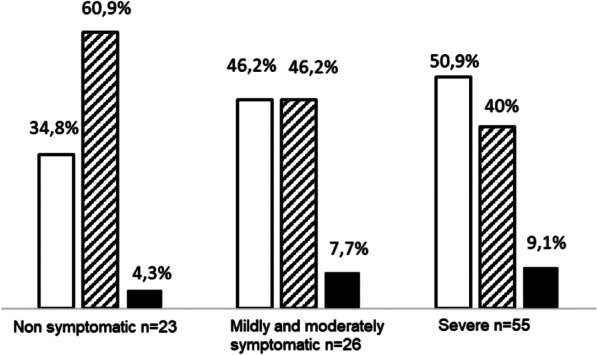
Frequency of the TaqI polymorphism genotypes, according to the clinical form in 104 Cuban patients suffered different clinical forms of COVID-19

Figure 1 shows the frequency of polymorphism genotypes in the three clinical forms, tt appeared in the lowest proportion in all three forms, and it increased with the severity of the disease.

Table 1 shows the number of patients studied according to their genotype for the TaqI polymorphism of the *VDR* gene and the possibility that they presented a given clinical form (symptomatic or asymptomatic) compared to the risk of presenting another (OR). It was observed that those who presented the Tt genotype had the highest OR among the asymptomatic forms [OR = 2.150, 95% CI: 0.835–5.541], while those who presented the tt genotype showed a highest probability among the symptomatic forms [OR = 2.081, 95% CI: 0.243–17.842].

**Table 1:** Cuban patients studied (*n* = 104), according to their genotype for the TaqI polymorphism of the VDR gene and the possibility of a clinical form -whether non symptomatic or symptomatic- compared to the risk of presenting another clinical forms

Clinical forms	Genotypes for the TaqI polymorphism					
	tt		Tt		TT	
	No	OR (IC = 95%)	No	OR (IC = 95%)	No	OR (IC = 95%)
Symptomatic (*n* = 81)	7	2.081 [0.243–17.842]	34	0.465 [0.180–1.198]	40	1.829 [0.699–4.788]
Non symptomatic (*n* = 23)	1	0.481 [0.056–4.120]	14	2.150 [0.835–5.541]	8	0.547 [0.209–1.431]

OR: *odds ratio*

CI: confidence interval

When doing a similar analysis -only regarding the severity in the symptomatic forms- it was observed that those who had a tt genotype were more likely to develop severe forms [OR = 1.200, 95% CI: 0.217–6.638], rather than mild and moderate symptoms [OR = 0.833, 95% CI: 0.151–4.610].

The tt genotype of this polymorphism -corresponding to the homozygous for the mutated allele- is the least frequent in human populations, including the normal Cuban population, as observed in this research [12, 19]. It was related to a greater probability of developing rather symptomatic forms, and even to the probability of developing severe forms. In opposition, it was observed that individuals with the Tt genotype were more likely to develop asymptomatic forms of the disease. It could, therefore, be interpreted that the tt genotype constitutes a risk factor, and the Tt a protective condition. However, since the confidence interval for all ORs contains value one, these inferences cannot be drawn. This can be explained by the small sample size of this study, which was a major limitation of this research work. However, the results are congruent with the observations regarding the role of Vitamin D, and thus its receptor, in the clinical development of this disease. [20].

Undoubtedly, there are other risk factors related to COVID-19 such as: age, comorbidities, and even other polymorphisms. According to this research, older patients were likely to present more severe forms [2, 20]. However, the analyzed results, although preliminary, merit further research, which could serve as a basis for the development of preventive strategies for COVID-19.

## Conclusion

There are signs of association between the risk of developing COVID-19 and the genotypes of the TaqI polymorphism of the *VDR* gene in the studied Cuban patients.

## Data Availability

The datasets used and/or analyzed during the current study are available from the corresponding author on reasonable request.

## Abbreviations

ACE2: Angiotensin Converting Enzyme 2
CI: Confidence interval
DNA: Deoxyribonucleic acid
EDTA: Ethylenediaminetetraacetic acid
OR: Odds ratio
PCR: Polymerase chain reaction
SARS-CoV-2: Severe acute respiratory syndrome coronavirus 2
SPSS: Statistical Package for Social Sciences
TaqI enzyme: Restriction enzyme isolated from the bacterium Thermus aquaticus
VDR: Vitamin D Receptor
